# Congenital syphilis in a preterm newborn with gastrointestinal disorders and postnatal growth restriction

**DOI:** 10.1186/s13052-022-01404-5

**Published:** 2022-12-29

**Authors:** Gregorio Serra, Maurizio Carta, Maria Rita Di Pace, Eleonora La Sala, Ettore Piro, Sergio Salerno, Ingrid Anne Mandy Schierz, Alessia Vassallo, Mario Giuffrè, Giovanni Corsello

**Affiliations:** Department of Health Promotion, Mother and Child Care, Internal Medicine and Medical Specialties “G. D’Alessandro”, University of Palermo, Palermo, Italy

**Keywords:** Congenital syphilis (CS), Prematurity, Gastrointestinal symptoms, Growth delay, Case report

## Abstract

**Background:**

Congenital syphilis (CS) depends on the placental transmission of *Treponema pallidum* (TP) spirochetes from an infected mother to fetus during pregnancy. It shows a wide clinical variability with cutaneous and visceral manifestations, including stillbirths, neonatal death, and asymptomatic cases. Preterm infants with CS may have more severe features of disease than the term ones, due to the combined pathogenic effect of both CS and prematurity.

**Case presentation:**

We report on a female preterm (32^+6^ weeks of gestation) newborn showing most of the typical CS manifestations, in addition to gastrointestinal disorders including feeding difficulties, colon stenosis and malabsorption leading to postnatal growth restriction. The mother resulted positive at the syphilis screening test of the first trimester of pregnancy, but she did not undergo any treatment. At birth, our newborn was VDRL positive (antibody titer four times higher compared to the mother), and she was treated with intravenous benzathine benzylpenicillin G for 10 days (50,000 IU/Kg three times per day). Poor tolerance to enteral nutrition (abdominal distension, increased biliary type gastric secretions) was observed. A barium enema X-Ray identified a colon stenosis within the descending tract. However, the poor general conditions due to a concurrent fungal sepsis did not allow to perform any surgical procedure, and a conservative approach with total parenteral nutrition was started. The following evolution was marked by difficulties in enteral feeding including refusal of food and vomiting, to which also contributed the neurological abnormalities related to a perinatal asphyxia, and the affective deprivation for the physical absence of the mother during hospitalization. At 5 months of age, after the introduction of an amino acid-based formula (Neocate LCP Nutricia ®), an improvement of enteral feeding was observed, with no further and significantly decreased episodes of abdominal distension and vomiting respectively, and regular stool emission. A psychological support offered to the family allowed a more stable bond between the mother and her baby, thus providing a significant additional benefit to food tolerance and growth. She was discharged at 5 months of age, and included in a multidisciplinary follow-up. She at present shows global growth delay, and normal development apart from mildly increased tone of lower limbs.

**Conclusions:**

Our report highlights less common clinical CS manifestations like gastrointestinal disorders including feeding difficulties, colon stenosis and malabsorption leading to postnatal growth delay. Moreover, it underlines how prematurity may worsen the clinical evolution of such congenital infection, due to the additional pathogenic effect of possible associated diseases and/or conditions like sepsis, hypoxic/ischemic injury, or use of drugs. CS may be observed also in high-income countries, with high rates of antenatal screening and availability of prenatal treatment. A multidisciplinary network must be guaranteed to the affected subjects, to ensure adequate care and improve the quality of life for patients and their families.

## Background

Congenital Syphilis (CS) results from vertical transmission of *Treponema pallidum* (TP) from infected untreated pregnant women to the embryo and/or fetus. The infection can also be transmitted during delivery, if active lesions within the vaginal canal are present. Syphilis in pregnancy is among the main causes of stillbirth globally, and may also result in neonatal death, prematurity, low birth weight, and variable malformative lesions in newborns and babies [1]. Severity of clinical manifestations depends on time of contagion of the pregnant woman, and neonatal mortality and morbidity are higher if transplacental transmission occurs during the first and second trimester [2, 3]. Based on time of presentation, CS may be classified into early (< 2 years of life) and late disease (> 2 years of life). The first one develops by 2 to 8 weeks of life, and its most common symptoms include hepatosplenomegaly, lymphadenopathy, mucocutaneous lesions, osteochondritis, pseudoparalysis, edema, rash, hemolytic anemia, thrombocytopenia, rhinitis or “snuffles”. If not treated about 40% of infected neonates will develop late CS, characterized by the Hutchinson’s triad (interstitial keratitis, Hutchinson’s teeth and eighth-nerve deafness), and neurodevelopmental delay [3, 4]. Literature data reported recently increasing incidence rates in Europe, with 1.6 cases per 100,000 live births estimated in 2018 [5], as well as in Italy with 5 cases per 100,000 live births observed between 2018 and 2022 [6]. The main methods for screening, diagnosis, and monitoring of syphilis are serological tests. Treponemal ones are more sensitive in early infection. A neonatal antibody titer 4 times higher than the maternal one is suggestive for neonatal infection diagnosis, which may be confirmed by immunoblotting [7].

World Health Organization (WHO) recommends antenatal screening during the first and third trimester of pregnancy [1]. Identification and treatment of congenital syphilis at birth or within 3 months of life prevent late sequelae. Aqueous crystalline or procaine penicillin are the treatments of choice for CS. Hereby, we report on an Italian female preterm newborn affected by CS showing most of the typical clinical manifestations of congenital infection, in addition to uncommon gastrointestinal disorders, including feeding difficulties, colon stenosis and malabsorption leading to postnatal growth delay, which needed a particularly complex diagnostic and therapeutic management.

## Case presentation

A female preterm newborn, second child of Italian non-consanguineous parents, was delivered at 32^+6^ weeks of gestation (WG) by caesarean section. The mother resulted positive at the screening test for syphilis (TPHA and VDRL) during the first trimester of pregnancy, but she did not undergo any treatment due to miscommunication by the couple of anamnestic data and misinterpretation by obstetricians of laboratory results. The other serological tests for TORCH agents were negative. Current pregnancy was also marked by gestational diabetes, requiring diet treatment. At birth, anthropometric measurements were as follows: weight 1590 g (24^th^ centile, -0.71 standard deviations, SD), length 41 cm (24^th^ centile, -0.71 SD) and occipitofrontal circumference (OFC) 29 cm (24^th^ centile, -0.7 SD). Apgar scores were 1 and 7, at 1 and 5 min respectively. Postnatally, she was intubated and mechanically ventilated for about 72 h. Microbiological examinations documented positive nontreponemal test for syphilis (1:256, four times higher compared to 1:64 of the mother, tested again two and five months after birth, and showing a progressive decrease of the antibody titer till 1:4; Table 1), Immunoblotting IgM, IgG, IgA antibodies against TP, and PCR real time of *T*. *pallidum* DNA performed on peripheral blood.

**Table 1:** Microbiological (treponemal and non-treponemal tests, Immunoblotting, *T. pallidum* DNA PCR) profiles of our patient and her mother during pregnancy and after delivery

	TPHA	VDRL (RPR)	Liaison (UA/ml)	Immunoblotting IgG/IgM	*T. pallidum* DNA PCR
**Mother**					
8^th^ week of pregnancy	Negative	Negative			
9^th^ week of pregnancy	1:280	Positive (not quantified, performed in a different analysis center)	46.8		
12^th^ week of pregnancy	1:640	Positive (not quantified, performed in a different analysis center)			
20 days after delivery	1:40,960	1:64			
2 months and 16 days after delivery and the administration of three I.M. doses (1,200,000 IU) of Benzathine Benzylpenicillin		1:16	45.4	Present/Present	
5 months after delivery		1:2			
**Daughter**					
Day 1		1:256	43.4	Present/Present	Positive
2 months and 12 days		1:64	13.0	Present/Present	
5 months and 18 days		1:4	20.7	Present/Absent	

*I.M.* Intramuscular, *TPHA Treponema Pallidum* Haemagglutination Assay, *VDRL* Venereal Disease Reference Laboratory, *RPR* Rapid Plasma Reagin

**Fig. 1:**
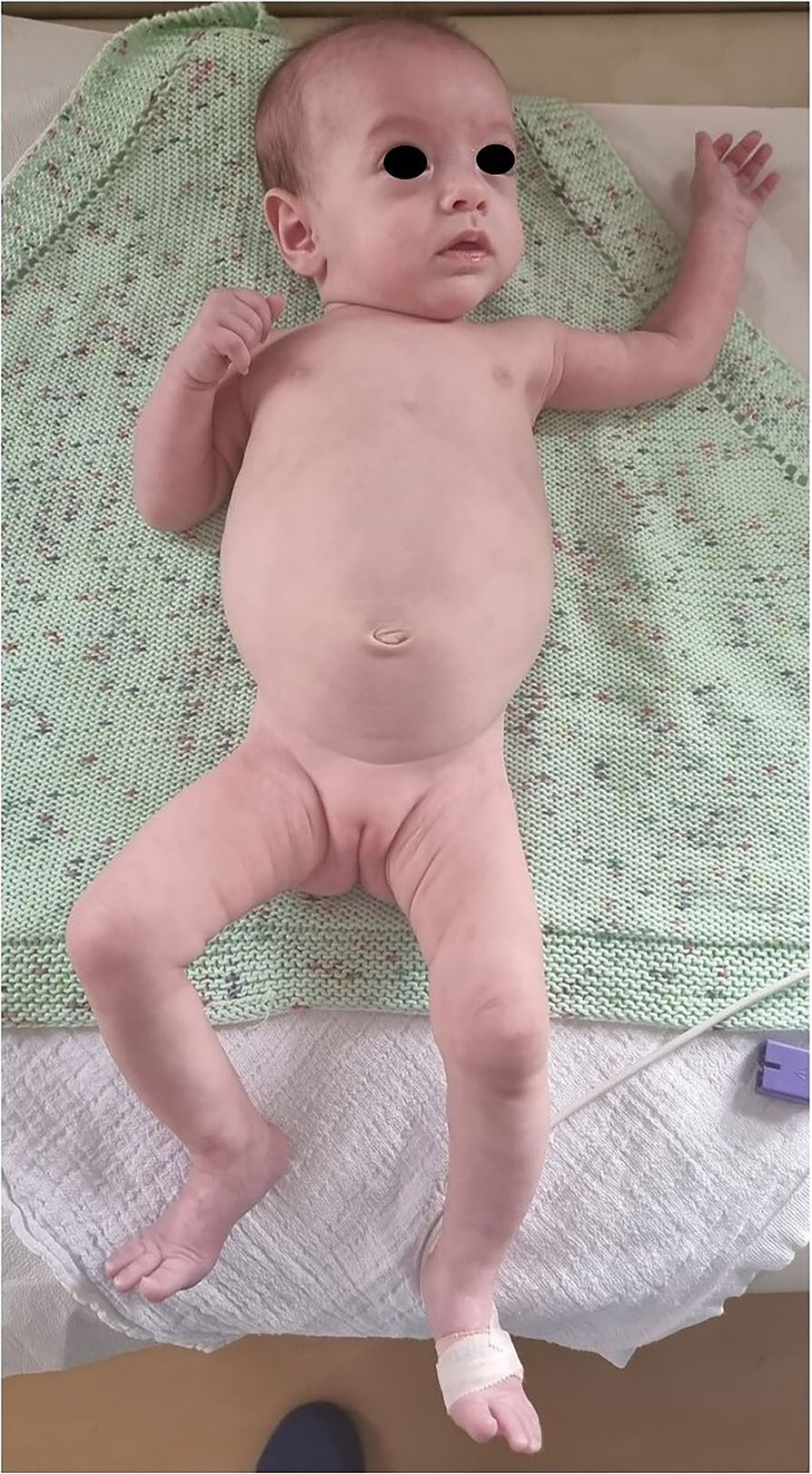
Our patient at age 5 months: frontal bossing, depressed nasal bridge with rounded tip, thin upper lip, small chin, and signs of malabsorption including pale skin, sparse hair and eyebrows, globose abdomen, dystrophy of the limbs

Therefore, an intramuscular single dose of benzathine penicillin G (BPG) 50,000 IU/Kg was administered. During the first two weeks of life the clinical course was marked by anemia and thrombocytopenia, which required red blood cell and platelet transfusions respectively. Due to poor tolerance to enteral feeding, with the onset of gastrointestinal manifestations including globose abdomen, increased gastric secretions and cholestatic jaundice, she was referred on day 14 of life from the birthing center to our Neonatal Intensive Care Unit. At admission, physical examination showed pale skin, frontal bossing, depressed nasal bridge with rounded tip, “snuffles” (i.e., syphilitic rhinitis), thin upper lip, small chin, hepatosplenomegaly, abdominal distension (Fig. 1). Ophtalmological evaluation disclosed bilateral keratitis, for which a local treatment with desametasone and atropin eye drops was started. Moreover, a therapy with intravenous (iv) BPG (50,000 IU/Kg three times per day) was administered for 10 days. Abdominal X-Rays ruled out intestinal malrotation and/or acute abdomen, and a conservative approach continuing total parenteral nutrition was undertaken. Head ultrasound (US) showed lateral ventricles dilation, diffuse periventricular hyperchogenicity with millimetric cavitated lesions, also within choroid plexuses and the germinative matrix, and increased resistive index (RI 0.90). Heart US disclosed hypertrophic cardiomyopathy and interventricular septum. Abdominal US revealed hepatosplenomegaly (longitudinal diameter of the spleen 5 cm). Skeletal X-ray did not evidence abnormalities of bones or joints. Laboratory tests documented increased levels of transaminases, gamma-glutamyl transferase (GGT) and direct bilirubin according to hepatopathy, and low values of serum albumin and total proteins compatible with malabsorption, which progressively normalized within the following weeks (Table 2).

**Table 2:** Serum hepatic and protidemic profiles of present patient

	2^nd^ week	2^nd^ month	3^rd^ month	4^th^ month	5^th^ month
AST (IU/L, n.v. 0–84)	661	543	252	134	71
ALT (IU/L, n.v. 0–60)	451	350	246	88	46
GGT (IU/L, n.v. 5–36)	126	114	93	89	17
TSB/D (mg/dl, n.v. 122–469)	5.5/5.2	3.7/2.9	2.2/1.5	1.4/1.06	< 0.15/0
Albumin (g/L, n.v. 38–54)	27	23	35	42	37
Total proteins (g/L, n.v. 66–87)	45.8	41.4	56.6	59.4	55.5

*ALT* Alanine aminotransferase, *AST* Aspartate aminotransferase, *GGT* Gamma-glutamyl transferase, *TSB/D* Total serum bilirubin/direct bilirubin, *n.v.* Normal values

**Fig. 2:**
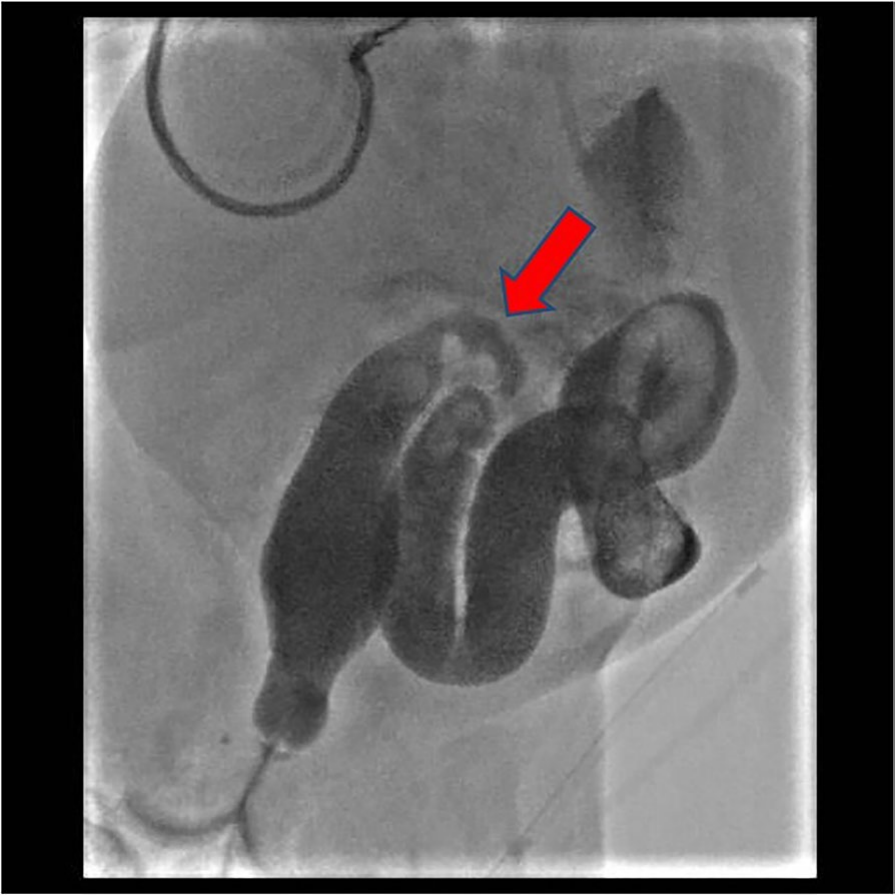
Contrast barium enema: dolichosigma with regular contrast progression until a stenosis (showed by the arrow), spanning about 2.7 cm within the distal descending tract

Subsequently, after the reintroduction of enteral nutrition, a new worsening of the abdominal picture with the appearance of bile-stained vomiting and constipation occurred. Further instrumental investigations were, then, carried out. X-ray study of the digestive tract showed good gastric filling and opacification of duodenum and first jejunal loops, while the double-contrast barium enema examination documented dolichosigma with regular contrast progression until a colic stenosis, spanning approximatively 2.7 cm within the distal descending tract (Fig. 2).

The concomitant occurrence of a fungal sepsis, sustained by *Candida parapsilosis*, did not allow the patient to undergo any surgical procedure. However, after iv antimycotic therapy with liposomal amphotericin B and micafungin and continuation of parenteral nutrition (performed for about 3 months) a clinical improvement was noted, and enteral feeding was introduced again. Nonetheless, minor gastrointestinal disorders persisted with frequent regurgitation and prolonged inconsolable crying during feedings, but without further episodes of relevant abdominal distention and/or constipation. In addition, actually, at age 3 months and 28 days (corresponding to 2 months and 6 days of corrected age) neurological findings were axial hypotonia and extensor hypertonia of the limbs, poor spontaneous movements characterized by sturtles with crampiform features, along with decreased coordination between sucking and deglutition. Moreover, the mother was not available to visit the baby for the first three months of hospitalization. A psychological assessment highlighted a problematic familiar background with conflicting relationships, also due to the sense of guilt for infecting the baby and not being treated during pregnancy.

At age 5 months (the last month of the hospital stay), after the introduction of a free synthetic amino acid-based formula (Neocate LCP Nutricia ®), an improvement of feeding tolerance was noted, with decrease of the vomiting episodes and regular stool emission. Furthermore, the enrollment into an habilitation plan including logopedic treatment and a psychological support offered to the mother permitted the creation of a more stable and efficient bond with the daughter, providing thus an additional significant benefit to the gastrointestinal disorders and finally also to the growth rate. Multiorgan US follow-up evaluations evidenced a gradual and complete resolution of the abnormalities previously described, including encephalic lesions, hypertrophic cardiomyopathy and hepatosplenomegaly. Ophthalmic lesions disappeared as well, and topical treatment was stopped (already at age 2 months). Hip US identified normal ripening of the joints (1A Graf stage), and abdominal US ruled out a hypertrophic pyloric stenosis (thickness of 2 mm) [8, 9]. Hearing screening through transient-evoked otoacoustic emissions (TEOAEs) revealed normal results, as well as the hearing auditory brainstem response (ABR). Visual evoked potentials were also performed, and detected latency of the main components within the normal values for post-menstrual age, with less amplitude in the right hemisphere. The last days of the hospital stay were finally characterized by a bowel infection by *Clostridium difficile*, for which a treatment with oral vancomycin was administered for two weeks [10].

She was discharged at 5 months and 20 days, and included in a multidisciplinary (auxological, gastroenterological/surgical, neurodevelopmental, ophthalmological, audiological) follow-up. Currently, she is 6 months and 12 days old (4 months and 21 days of corrected age) and has, according to World Health Organization growth chart for neonatal and infant close monitoring [11], a severe global growth failure: weight 4230 g (< 0.4^th^ centile, -3.86 SD), length 57 cm (< 0.4^th^ centile, -2.96 SD), OFC 38 cm (< 0.4^th^ centile, -2.52 SD). Physical examination at present still shows signs of malabsorption (Fig. 1), despite a decrease of abdominal protrusion, the regularity of stool emission and the absence of other abnormalities or symptoms attributable to intestinal obstruction, allowing thus further instrumental investigations not to be performed. Neurodevelopmental assessment is presently normal in relational, behavioral and motor areas with normal righting reactions, apart a mild delay due to an increased passive tone of the lower limbs.

## Discussion and conclusions

Congenital syphilis is due to *Treponema pallidum* embryonal and/or fetal infection. Despite antenatal screening, CS incidence rate is increasing worldwide [5, 6, 12] and nowadays it remains one of the main causes of stillbirth and neonatal mortality. The risk of TP vertical transmission depends on the stage of maternal infection and increases at increasing gestational age, while severity of clinical manifestations is conversely higher if infection occurs during the first and second trimester [12].

CS is associated with miscarriage, stillbirth, nonimmune hydrops, and may lead to two different stages of clinical disease, known as early and late congenital syphilis. In early CS, clinical manifestations appear up to 2 years of age and include hepatosplenomegaly, lymphadenopathy, mucocutaneous lesions, osteochondritis, pseudoparalysis [13], edema, rash, hemolytic anemia, hepatitis, thrombocytopenia, rhinitis or “snuffles”. Conversely, in late CS the clinical signs, which involve dentition, eye, bones, central nervous system (CNS) and other organs (summarized in Table 3), occur after 2 years of age.

**Table 3:** Main clinical, laboratory and radiological findings of early and late CS (modified by Cooper JM et al., 2018 [12])

	**Early Congenital Syphilis**	**Late Congenital Syphilis**
**Signs and symptoms**	Preterm birth, stillbirth Nonimmune hydrops fetalis Intrauterine growth restriction/small for gestational age Hepatomegaly with or without jaundice Splenomegaly Skin rash Adenopathy (characteristically palpable epitrochlear nodes), rhinitis (snuffles) Mucus patch Condylomata lata Pseudoparalysis of Parrot Chorioretinitis, cataract Central nervous system: asymptomatic invasion, cranial nerve palsies, seizures	Dentition: Hutchinson’s teeth, Mulberry molars Eye: interstitial keratitis, healed chorioretinitis Eighth nerve deafness Rhagades Central nervous system: cognitive disability, hydrocephalus, seizures, optic nerve atrophy, juvenile general paresis, cranial nerve palsies Bone/Joint: frontal bossing, saddle nose deformity, protuberant mandible, short maxilla, high palatal arch, saber shin, sternoclavicular joint thickening (Higouménakis sign), Clutton’s joints
**Laboratory tests**	Anemia, thrombocytopenia Hypoglycemia Cerebrospinal fluid pleocytosis, elevated protein content Liver transaminitis and direct hyperbilirubinemia	
**Radiographic findings**	Bone abnormalities: periostitis, osteochondritis. Pneumonia alba	
**Other manifestations**	Nephrotic syndrome, pancreatitis, myocarditis, fever, gastrointestinal malabsorption, hypopituitarism (diabetes insipidus)	

Our patient presented with the typical features of early CS. Indeed, she was born preterm, and showed since the first days of life anemia, thrombocytopenia, rhinitis (“snuffles”, a nasal discharge rich of spirochetes [12]), hepatosplenomegaly, liver transaminitis, cholestatic jaundice, in addition to other less common manifestations like keratitis and gastrointestinal disorders including feeding refusal/vomiting, bowel stenosis and malabsorption, leading to subsequent growth restriction.

In early CS bowel is rarely involved, while the usually affected organs are kidneys, bones, liver, pancreas, spleen, eyes and skin [14]. The first cases of intestinal lesions associated to CS were described in 1939 by D'Aunoy (who histologically analyzed gut samples of patients affected by CS) [15, 16], and later confirmed by Bittencourt in 1979 [17]. The latter documented the mucosal lymphocytic infiltration in gut samples of affected subjects, and also showed that CS can be linked with a severe pancreatic fibrosis, leading to inspissated meconium and mechanical intestinal obstruction [14, 17]. CS can also be the cause of intestinal atresia or stenosis [16–19], as our case reveals. Such clinical manifestations are due to an intrauterine ischemic injury caused by syphilitic arteritis, or, as sustained by other Authors [18], by a syphilitic enterocolitis. Although rarely, intestinal obstruction may also be complicated by gastrointestinal (GI) bleeding, like rectal hemorrhage, owing to mucosal ulceration [20]. Lee HS et al. found a correlation between prematurity and the GI manifestations of CS. Indeed, they showed how meconium obstruction depends on syphilitic enterocolitis and pancreatitis, in addition to the immaturity of the enteric nervous system. The persistent cholestasis may be, as well, the result of both syphilitic hepatitis and immaturity of the newborn liver [21].

All these CS cases showing GI manifestations have been treated with a surgical approach, including resection of the stenotic or ulcerated intestinal regions, and packaging of a terminus-terminal anastomosis [15–21] (Table 4).

**Table 4:** Overview of the previous studies on congenital syphilis (CS) associated with gastrointestinal disorders (GID)

Reference	CS cases	Incidence of GID and clinical features	Histological patterns
D'Aunoy R et al.; Arch Pathol, 1939 [15]	230	1.3% of CS cases have shown an intestinal involvement	Histological study documented raised yellow bands encircling the ileum wall as gross evidence of syphilitic involvement of the intestine
Oppenheimer EH, Hardy JB; Johns Hopkins Med, 1971 [16]	11	The authors detailed the histopathologic lesions of the gastrointestinal tract that they found in 11 patients with congenital syphilis	Histological exam evidenced an intense fibroblastic infiltrate in the mucosa and a submucosal and mononuclear cell infiltration involved diffusely throughout the small bowel, and in a few cases the stomach, duodenum, and colon
Bittencourt AL et al.; Acta Med Port, 1979 [17]	13	The paper describes the histopathological lesions of the small and large bowel in 13 cases of congenital syphilis. This study includes 4 non-macerated stillbirths, 7 neonatal deaths and 2 infants who died at ages 2 and 16 months	Histological exam evidenced an inflammatory infiltrate, formed by histiocytes and plasmocytes in the mucosa and submucosa
Siplovich L et al.; J Pediatr Surgery, 1988 [18]	4	In 3 cases the obstruction was due to inspissated meconium, simulating the features of meconium ileus in one, meconium plug in the second, and associated with perforation of the terminal ileum in the third. The fourth patient had multiple ileal stenoses	The stenosis and the obstruction are due to bowel ischemia. The histological pattern showed syphilitic arteritis of the bowel wall
Heydenrych JJ et al.; S Afr Med J, 1988 [19]	1	A week-old baby presented with abdominal distension, small-bowel obstruction, bilious vomiting and a large left upper quadrant mass	Histopathological examination of a specimen of the mass confirmed the clinical diagnosis of gumma formation
Ajayi NA et al.; Pediatr Surg Int, 1999 [20]	1	In this case a VDRL-positive infant developed incomplete intestinal obstruction and rectal bleeding. He underwent ileal resection and primary end-to-end anastomosis with resolution of his symptoms	Terminal ileal inflammation and stenosis were demonstrated. Histopathological examination showed heavy plasmacytic infiltration of the lamina propria and submucosa with ulceration of the mucosa, consistent with syphilitic ileitis
Lee HS et al.; Clinical Case Report Medicine (Baltimore), 2020 [21]	1	A very low birth weight infant at 32 weeks of gestation presented with meconium obstruction, prolonged cholestatic jaundice with elevated liver enzymes, and disseminated intravascular coagulation with a bleeding diathesis, in addition to common or typical CS manifestations	Histological exam not performed

In our case the poor clinical conditions of the patient did not allow us to perform surgery, and then we opted for a non-invasive approach. Indeed, the newborn initially underwent total parenteral nutrition, and she was later gradually weaned to a full enteral nutrition, based on an amino acid formula [22]. Such feeding approach is recommended for cow’s milk protein intolerance and gastrointestinal protein malabsorption and motility issues, since it may reduce intestinal inflammation and immunopathologic alterations and, thus, also the risk of GI obstruction [22, 23] (Table 5).

**Table 5:** Specialized infant formulas suitable for patients with gastrointestinal disorders

Type of infant formula	Clinical indications
Lactose-free infant formulas	Lactose intolerance for congenital lactase deficiency (very rare) or transient lactose deficiency due to acute gastroenteritis (especially for malnourished infants)
Partially hydrolyzed cow’s milk formulas	Easily digested by reducing transit time and gastrointestinal distress for infants experiencing fussing, colic and constipation
Extensively hydrolyzed cow’s milk protein formulas (eHF)	Recommended for cow’s milk/soy protein intolerance and sensitivity and for gastrointestinal or hepatobiliary disease–related significant malabsorption
Amino acid formulas	Use warranted in infants who do not tolerate eHF as well as for diseases like protein malabsorption, gastrointestinal tract motility issues, short bowel syndrome, severe food allergies, and eosinophilic gastroenteropathies

The nutritional therapy, indeed, along with the antibiotic treatment for CS, the overcoming of the fungal and *Clostridium difficile* infections, and the progressive development of the immature bowel, permitted the improvement of both colic stenosis and malabsorption, and then a partial growth recovery [24, 25]. Actually, also the issue of the failure to thrive may recognize multiple pathophysiological mechanisms, related both with the intestinal lesions due to CS and with the feeding problems (food refusal, incoordination sucking/deglutition) linked in turn with the prematurity/birth asphyxia-related neurological abnormalities, and partly to the affective deprivation the baby suffered during the hospitalization [26, 27]. The latter likely contributed in causing the feeding refusal, since during the first months of the hospital stay our patient did not receive visits from her mother, who suffered from depression due to the sense of guilt she experienced for the transmission of the infection to her daughter during pregnancy. Effectively, it is well-known that mother-infant contact and early sucking in the *postpartum* period improve maternal sensitivity and child self-regulation. Specifically, the stimulation created by the contact between mother and child allows the baby to decrease anxiety, and to be less irritable and stressed [28]. Indeed, after having provided psychological support to the mother, in the last weeks of hospitalization she increased the time spent with her daughter. This permitted the creation of a stronger and more stable bond, resulting in the improvement of the patient’s emotional expressiveness. In fact, she was globally more relaxed and less irritable, including during feeding, and this gradually contributed to the improvement of nutritional issues and growth restriction [29].

In cases of pregnant women at risk and/or newborns with symptoms suggestive of CS, specific diagnostic tests must be carried out to confirm the diagnosis and quickly treat the infection [30]. Despite the availability of prenatal screening examinations and effective antenatal treatment, in recent years an increase of the incidence rate of congenital syphilis has been observed worldwide, including high-income countries as here reported [5, 6, 31]. According to our experience, in addition to the most frequent clinical manifestations of CS, neonatologists and pediatricians must be aware of less common clinical signs, such as gastrointestinal disorders, which need to be appropriately investigated and treated [32]. Moreover, our report underlines how prematurity may worsen the clinical evolution of CS, due to the additional pathogenic effect of associated diseases and/or conditions like sepsis, hypoxic/ischemic injury, or use of drugs [33–39].

Congenital syphilis may have, in addition to the potential injury to many different organs and systems, a relevant psychological impact for parents, who may feel guilty for the transmitted infection. Therefore, a multidisciplinary network involving neonatologists, obstetrics, microbiologists, infectivologists, radiologists, surgeons, pediatric neurologists and psychologists must be guaranteed to the affected subjects, to ensure adequate care and improve expectancy and quality of life for patients and their families [40–51].

## Data Availability

The datasets used and analyzed during the current study are available from the corresponding author on reasonable request.

## Abbreviations

BPG: Benzathine penicillin G
CNS: Central nervous system
CS: Congenital syphilis
GI: Gastrointestinal
iv: Intravenous
n.v.: Normal values
RI: Resistive index
TP: *Treponema pallidum*
TPHA: *Treponema pallidum* Haemagglutination assay
US: Ultrasound
VDRL: Venereal disease reference laboratory
